# Stoma‐Site Single‐Port Robotic Restorative Colorectal Surgery Using the da Vinci SP System: Technical Considerations and Early Outcomes

**DOI:** 10.1002/rcs.70241

**Published:** 2026-10-24

**Authors:** Hyeon Sik Jeong, Dong Jin Park, In Kyu Park, Songsoo Yang

**Affiliations:** ^1^ Department of Surgery Ulsan University Hospital Ulsan Korea; ^2^ Department of Surgery University of Ulsan College of Medicine Ulsan Korea

**Keywords:** adhesiolysis, da Vinci SP, reduced‐port surgery, robotic colorectal surgery, single‐port robotic surgery, stoma reversal

## Abstract

**Background:**

Restorative colorectal surgery after emergency stoma formation is challenging because of adhesions, altered anatomy, and pelvic dissection. We evaluated the technical feasibility of stoma‐site single‐port robotics restorative colorectal surgery using the da Vinci SP system.

**Methods:**

Six patients underwent restorative colorectal surgery through the previous stoma site between January 2023 and December 2024. The Institutional Review Board of Ulsan University Hospital approved this study (2026‐02‐018).

**Results:**

Five patients underwent Hartmann's reversal and one underwent completion proctocolectomy with ileal pouch–anal anastomosis. All procedures were completed robotically without open conversion; one required an additional port. Median operative time was 287.5 min and median blood loss was 25 mL. One intraoperative small‐bowel injury required resection, and one postoperative abscess required percutaneous drainage. No leakage, reoperation, readmission or mortality occurred.

**Conclusions:**

This approach was technically feasible in selected patients, but safety events highlight the difficulty of reoperative pelvic surgery. Further studies are required.

## 1 Introduction

Stoma reversal, including restoration of bowel continuity after Hartmann's procedure or end ileostomy, remains one of the most demanding operations in colorectal surgery [1]. Major technical challenges include dense and often unpredictable adhesions from the index surgery, distorted anatomy with limited working space and the need for precise pelvic dissection with intracorporeal reconstruction in the reoperative field. Nevertheless, reversal is often pursued because it relieves the physical and psychological burden of a stoma and may improve the quality of life. Therefore, optimising surgical strategies to reduce perioperative risks while facilitating recovery is clinically important.

Minimally invasive approaches can reduce wound morbidity and accelerate recovery compared with open surgery [2, 3, 4]. Single‐port laparoscopic reversal using the previous stoma site has shown outcomes comparable to those of conventional multi‐port laparoscopy in selected patients [5, 6, 7]. However, this approach is inherently limited by restricted triangulation and instrument crowding, and dense adhesions, particularly after emergency surgery, may lead to conversion and significant morbidity, limiting its broader applicability in reoperative settings. Thus, despite the evolution of minimally invasive surgery, maintaining technical control in hostile reoperative abdomens remains challenging.

Robotic platforms may mitigate these limitations by providing stable three‐dimensional vision and wristed instrumentation in confined spaces [8, 9, 10, 11]. The da Vinci single‐port (SP) system (Intuitive Surgical, Sunnyvale, CA, USA) enables a multichannel single‐site approach with flexible articulation through a single‐access cannula. Unlike conventional multiport systems, the SP platform allows multi‐instrument deployment and camera articulation through a single incision without additional trocar placement, potentially improving ergonomics while minimising abdominal wall trauma. In theory, this configuration may allow controlled initial entry through the prior stoma site, stepwise adhesiolysis, and intracorporeal reconstruction while minimising additional abdominal incisions.

We describe our initial experience with a stoma‐site SP robotic approach and evaluate its technical feasibility and early perioperative outcomes in restorative colorectal surgery.

## 2 Materials and Methods

### 2.1 Patients and Study Design

A retrospective review was performed of six patients who underwent restorative colorectal surgery using the da Vinci SP system via the previous stoma site between January 2023 and December 2024 at Ulsan University Hospital. Demographic and clinical data, including age, sex, body mass index (BMI), American Society of Anaesthesiologists (ASA) score, primary disease, operative findings, and postoperative course, were collected from electronic medical records. All patients had previously undergone emergency surgery for colorectal pathology.

This study was designed as a technical feasibility study of stoma‐site restorative colorectal surgery using the da Vinci SP platform rather than a procedure‐specific study of Hartmann's reversal alone. One patient who underwent completion proctocolectomy with ileal pouch–anal anastomosis (IPAA) was included because the procedure shared the same access strategy and core technical steps of pelvic dissection and intracorporeal reconstruction through the stoma‐site SP approach.

Patients were considered eligible for the SP stoma‐site approach if they were clinically stable, had no evidence of ongoing intra‐abdominal sepsis, and had a mobilisable stoma segment suitable for single‐site entry. Patients who had undergone diversion‐only stoma formation without colorectal resection were excluded. Cases with diffuse peritonitis, haemodynamic instability, or an inability to safely establish pneumoperitoneum through the stoma site were also excluded. This study was approved by the Institutional Review Board of Ulsan University Hospital (2026‐02‐018). The requirement for informed consent was waived because of the retrospective study design.

### 2.2 Preoperative Evaluation and Setup

The preoperative workup included routine laboratory testing, abdominopelvic computed tomography and colonoscopic evaluation of the distal segment when feasible. Mechanical bowel preparation and intravenous antibiotics were administered according to institutional protocols. The interval between the index surgery and reversal was determined based on clinical recovery, resolution of intra‐abdominal inflammation, and multidisciplinary assessment.

### 2.3 Surgical Technique

Before initiating this stoma‐site approach, the operating surgeon had substantial experience with minimally invasive colorectal surgery, conventional robotic surgery, and the da Vinci SP platform. This prior experience was considered important because the procedure requires adhesiolysis in a reoperative abdomen, pelvic dissection, intracorporeal reconstruction, and management of limited instrument triangulation through a single access route.

After induction of general anaesthesia, all patients were placed in the lithotomy position with their arms adducted. The preoperative abdominal appearance, including the previous stoma site and midline laparotomy scar, was documented before preparation and draping (Figure 1a). The previous stoma was circumferentially mobilised, and the bowel was transected at the mucocutaneous junction using a linear stapler (Figure 1b,c). When a circular stapled anastomosis was planned, an anvil was secured to the proximal segment before reintroduction into the abdominal cavity (Figure 1d). The stoma‐site fascial opening was prepared for single‐site access, and a finger sweep was used to assess peristomal adhesions and create a safe pocket for port insertion when necessary (Figure 1e).

**FIGURE 1:**
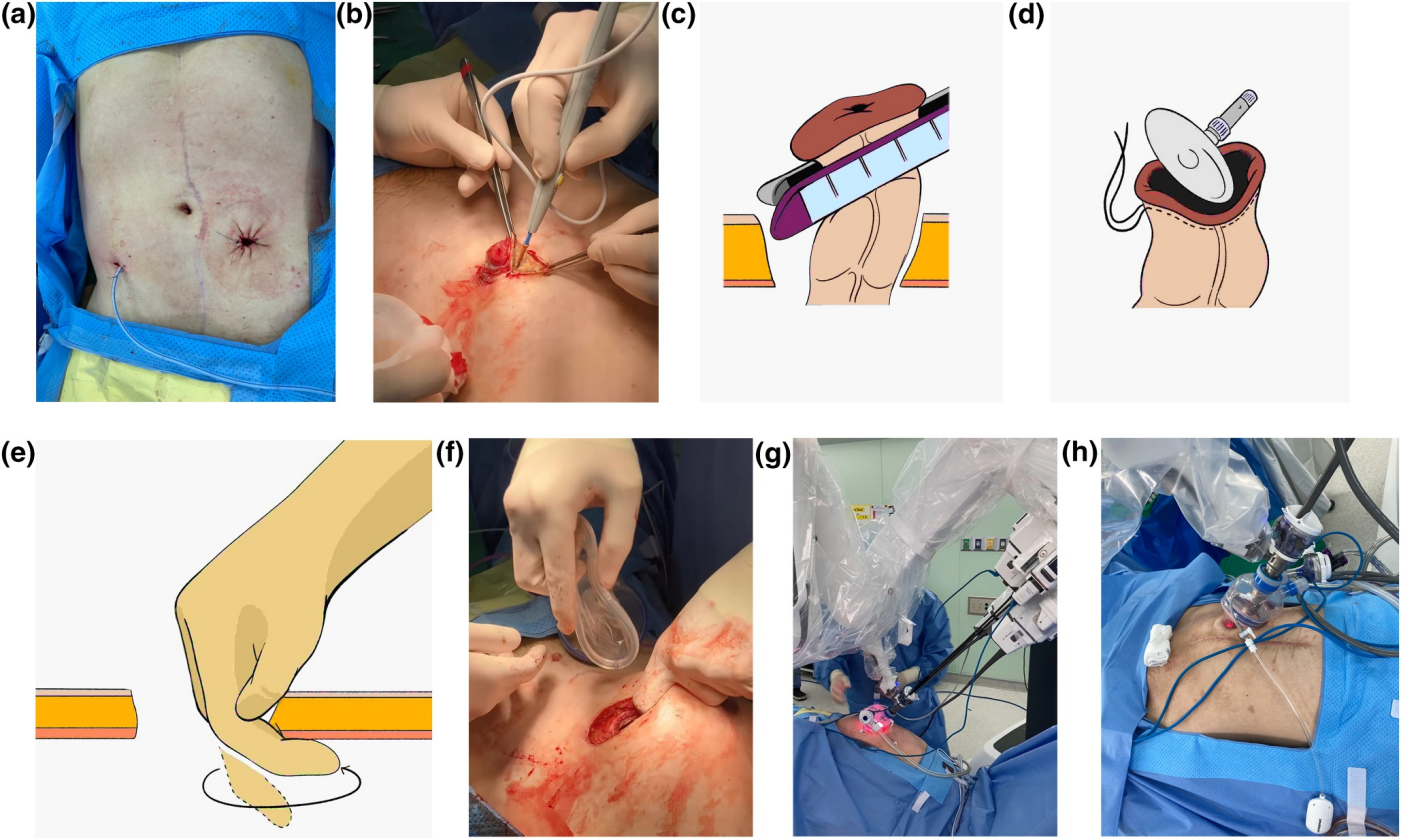
Preparation of the previous stoma site for single‐port robotic access. (a) Pre‐operative abdominal appearance showing the previous stoma site and midline laparotomy scar from prior emergency colorectal surgery. (b) Circumferential mobilisation of the previous stoma. (c) Transection of the bowel at the mucocutaneous junction using a linear stapler. (d) Anvil placement in the proximal bowel segment for circular stapled anastomosis. (e) Finger sweep and preparation of the stoma‐site fascial opening for safe port insertion. (f) Placement of the multi‐instrument single‐port device at the previous stoma site. (g) Docking of the da Vinci SP system directly onto the stoma‐site platform. (h) Instrument configuration through a single SP cannula.

A multi‐instrument single‐port device (Lapsingle Vision SP, Sejong Medical, South Korea) was mounted at the stoma site to establish the pneumoperitoneum (Figure 1f). After insufflation and Trendelenburg positioning, the da Vinci SP system was positioned on the patient's right side and docked directly onto the stoma‐site platform (Figure 1g). Standard instruments (monopolar curved scissors, fenestrated bipolar forceps and a round‐tooth retractor) were used through a single cannula (Figure 1h).

Adhesiolysis was performed as needed to develop the working space and mobilise the remnant colon and/or small bowel (Figure 2a,b,c). Dissection proceeded in a stepwise manner to maintain a clear operative field and minimise bowel injury in densely adherent areas. No additional skin incisions were made in routine cases, and all dissections were performed through the stoma‐site access.

**FIGURE 2:**
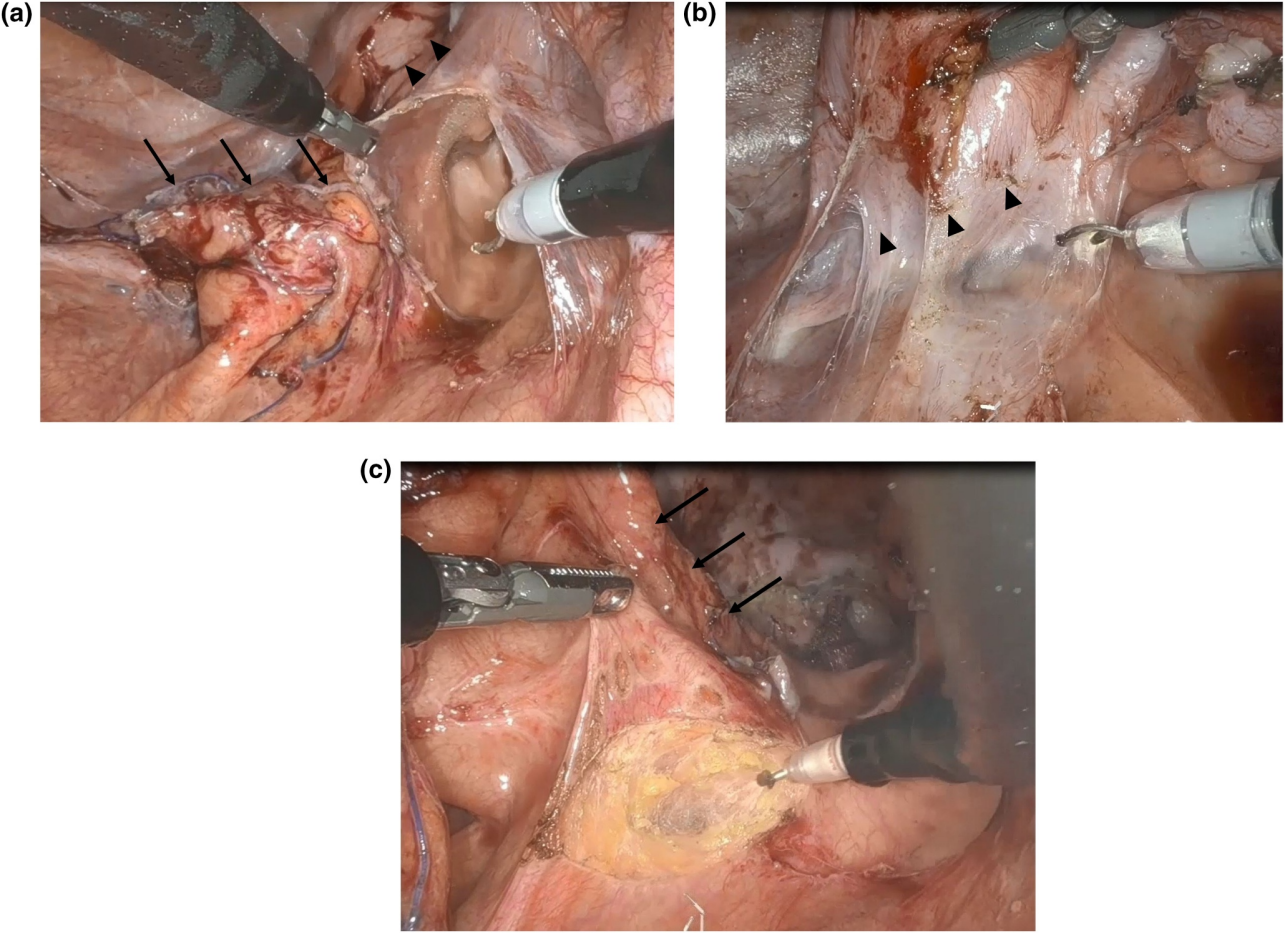
Adhesiolysis and rectal stump dissection under the SP robotic platform. Arrows: rectal stump. Arrowheads: uterus. (a) Adhesiolysis and initial identification of the rectal stump. (b) Dissection of the rectal stump from the uterus in a confined reoperative pelvis. (c) Complete mobilisation and exposure of the rectal stump. The SP robotic platform enabled stable visualisation and precise dissection during pelvic adhesiolysis through the stoma‐site access.

After mobilisation, bowel transection was performed using a laparoscopic linear stapler introduced through a single‐port platform (Figure 3a). One patient required an additional port for stapler access. Intracorporeal anastomosis was completed either by double‐stapling with a transanal circular stapler (Figure 3b,c) or by an isoperistaltic side‐to‐side stapled anastomosis using a linear stapler, depending on the intraoperative anatomy. In patients requiring IPAA, the pouch was constructed extracorporeally before completion of the anastomosis.

**FIGURE 3:**
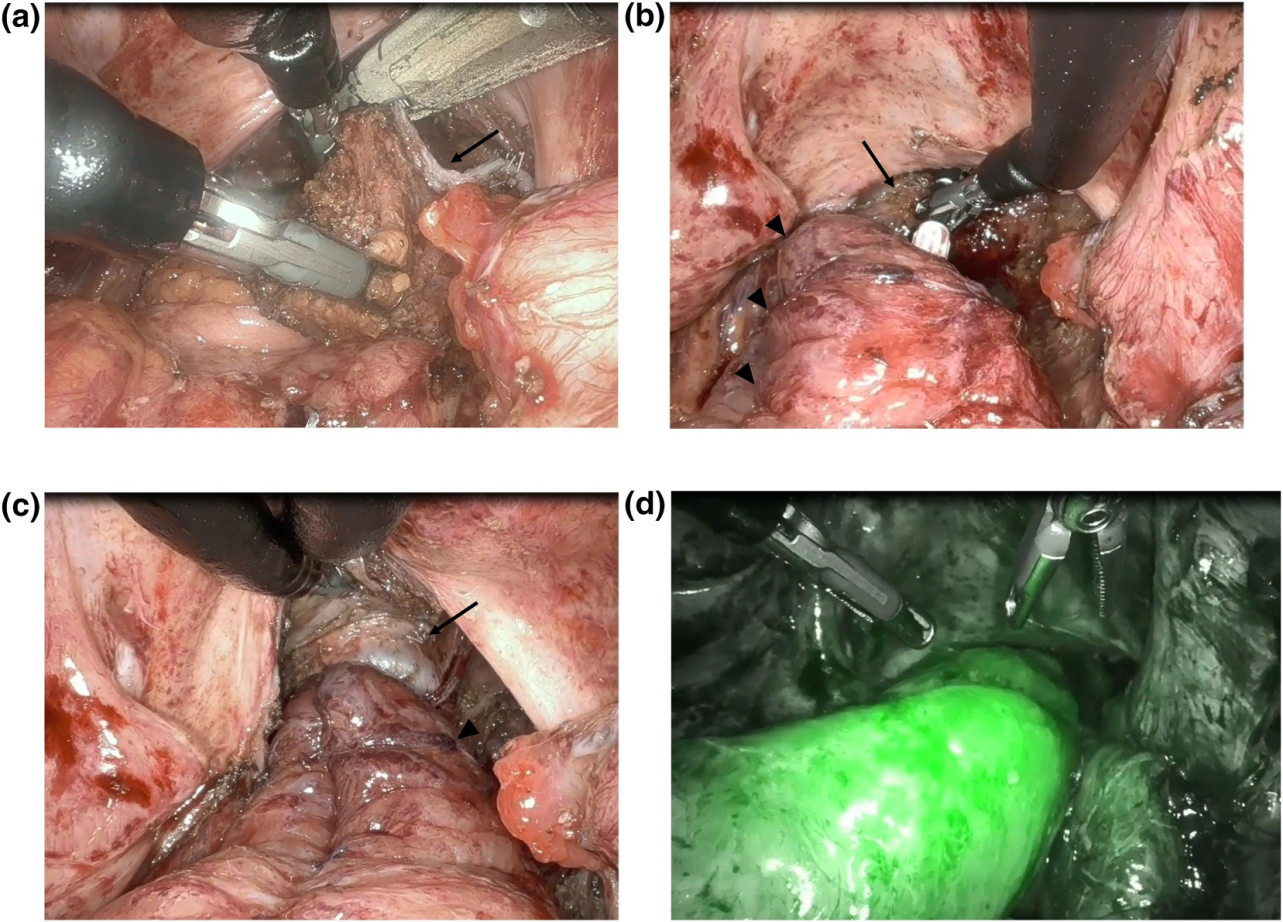
Intracorporeal reconstruction under the SP robotic platform. Arrows: ileal pouch–anal anastomosis (IPAA). Arrowheads: Distal rectal cuff (a) Resection of the remnant rectum using a linear stapler introduced through an assistant port. (b, c) Double‐stapling colorectal anastomosis using a transanal circular stapler after robotic mobilisation. (d) Perfusion assessment of the anastomotic site using indocyanine green fluorescence under near‐infrared imaging.

After reconstruction, perfusion of the anastomotic site or pouch was assessed using indocyanine green fluorescence under near‐infrared imaging (Figure 3d). The stoma‐site incision was closed in layers with fascial repair followed by purse‐string skin closure, leaving the previous stoma site as the main access wound (Figure 4). When a new diverting ileostomy was required, stoma reconstruction was performed through the same site.

**FIGURE 4:**
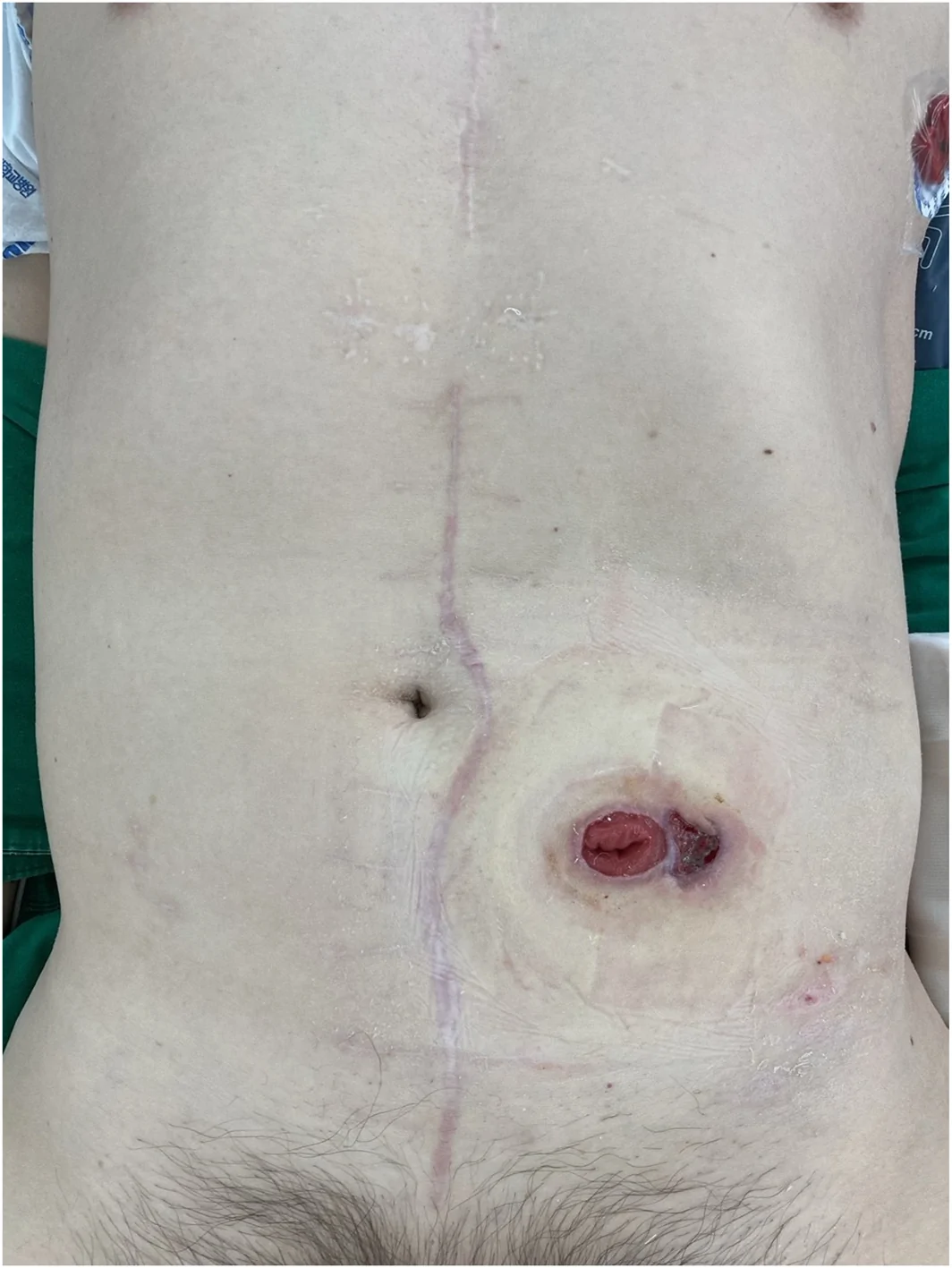
Closure of the stoma‐site incision. Final wound appearance after layered fascial closure and purse‐string skin closure of the stoma‐site access wound.

### 2.4 Postoperative Management and Follow‐Up

Patients were managed under an enhanced recovery protocol. Diet was advanced after the return of bowel function. Follow‐up included clinical examination and computed tomography within 3 months postoperatively. Postoperative complications were graded according to the Clavien–Dindo classification, and 30‐day outcomes were systematically reviewed.

### 2.5 Operative Metrics

Operative time was defined as the interval from skin incision to skin closure. Console time was defined as the time during which the operating surgeon performed the procedure at the robotic console.

### 2.6 Statistical Analysis

Given the small sample size, only descriptive statistics were used. Continuous variables are presented as median (range), and categorical variables as number (%). Statistical analyses were performed using the latest version of R available at the time of analysis (version 4.5.2; R Foundation for Statistical Computing, Vienna, Austria).

## 3 Results

Six patients (three men and three women) underwent SP robotics restorative colorectal surgery via the previous stoma site (Table 1). The median age was 59 years (range, 19–78), and the median BMI was 22.7 kg/m^2^ (range, 20.2–29.2). Five patients (83%) had ASA class III status and multiple comorbidities. The median interval between the index operation and restoration was 160 days (range, 110–520). Five patients underwent Hartmann's reversal, and one underwent completion proctocolectomy with ileal pouch‐anal anastomosis (IPAA).

**TABLE 1:** Baseline characteristics.

Case	Age (years)	Sex	BMI (kg/m^2^)	ASA class	Previous diagnosis	Previous operation
A	78	M	29.2	3	Ischaemic colitis	Hartmann's procedure
B	44	M	24.3	2	Perforated sigmoid diverticulitis	Hartmann's procedure
C	69	F	20.9	3	Ischaemic colitis	Hartmann's procedure
D	76	F	20.3	3	Sigmoid colon perforation	Laparoscopic Hartmann's procedure
E	19	F	20.1	3	UC with transverse colon perforation	Total colectomy with end ileostomy
F	49	M	24.9	3	Traumatic descending colon transection	Hartmann's procedure

Abbreviations: ASA, American Society of Anaesthesiologists; BMI, body mass index; UC, ulcerative colitis.

All procedures were initiated at the previous stoma site using the SP platform. All cases were completed robotically without open conversion. One patient required an additional port for stapler introduction. Detailed perioperative outcomes are summarised in Table 2.

**TABLE 2:** Perioperative outcomes.

Case	Interval (days)	Procedure	Additional port	Anastomosis	OP time (min)	Console time (min)	EBL (mL)	Transfusion	Intraoperative complications	Postoperative complications (CD)	LOS (days)
A	520	SP‐HR	No	Double stapling	270	210	100	No	No	No	6
B	110	SP‐HR	Yes	Double stapling	305	190	30	No	No	No	6
C	117	SP‐HR	No	Double stapling	270	162	10	No	No	Intra‐abdominal abscess (CD IIIa)	13
D	203	SP‐HR + SBR	No	Double stapling	600	459	400	Yes	Small bowel injury (R&A)	No	10
E	116	SP‐TPC + IPAA	No	Double stapling	340	253	20	No	No	No	4
F	210	SP‐HR	No	Isoperistaltic side‐to‐side anastomosis	135	98	5	No	No	No	2

Abbreviations: CD, Clavien‐Dindo classification; EBL, estimated blood loss; HR, Hartmann's reversal; IPAA, ileal pouch‐anal anastomosis; LOS, length of stay; OP, operation; R&A, resection and anastomosis; SBR, small bowel resection; SP, Single‐port; TPC, total proctocolectomy.

The median operative time was 287.5 min (range, 135–600), and the median console time was 200 min (range, 98–459). The median estimated blood loss was 25 mL (range, 5–400). One intraoperative small‐bowel injury occurred during adhesiolysis in a patient with dense adhesions after previous emergency surgery. The injury was recognized immediately and managed intraoperatively by segmental small‐bowel resection with primary anastomosis. This patient also required intraoperative transfusion. There were no open conversions or intraoperative deaths.

Postoperatively, one patient developed an intra‐abdominal abscess requiring percutaneous drainage and antibiotics, corresponding to Clavien–Dindo grade IIIa. No patient experienced anastomotic leakage, wound infection, stoma‐site incisional complications, or postoperative mortality. The median time to soft diet was 5 days (range, 1–12 days). The median hospital stay was 6 days (range, 2–13). One prolonged hospitalisation was due to institutional isolation for scabies rather than surgical morbidity. There were no 30‐day readmissions or deaths. Follow‐up imaging within 3 months revealed no additional complications.

Postoperative pain was generally well controlled, with peak Numeric Rating Scale (NRS) pain scores ranging from 5 to 7 (median, 6). Five patients required a single postoperative patient‐controlled analgesia (PCA) device, whereas one patient required replacement of the PCA because of persistent pain associated with an intra‐abdominal abscess. Overall, the median rescue analgesic requirements were 9 doses of tramadol and 4 doses of pethidine, although the patient with the postoperative abscess required increased analgesic support during conservative management.

## 4 Discussion

This study describes our initial experience with stoma‐site restorative colorectal surgery using the da Vinci SP system. Restorative procedures after emergency colorectal resection are technically demanding because of dense adhesions and deep pelvic work and often require additional abdominal incisions or open conversion. In six selected patients who had previously undergone emergency colorectal resection, all procedures were completed robotically through the previous stoma site without open conversion, and no mortality occurred. One patient developed a Clavien–Dindo IIIa intra‐abdominal abscess requiring percutaneous drainage, and one patient sustained an intraoperative small‐bowel injury during adhesiolysis requiring resection and anastomosis. Overall, these early results suggest that an SP robotic approach via the previous stoma site is technically feasible for restorative colorectal surgery in a reoperative setting, with most cases completed through the stoma‐site access alone.

Although five of the six patients underwent Hartmann's reversal, one patient underwent completion of proctocolectomy with IPAA. These procedures differ in anatomical complexity, pelvic workload, and expected functional outcomes. Therefore, the present study was framed not as a procedure‐specific analysis of stoma reversal, but as an early technical evaluation of a single‐access restorative colorectal strategy using the previous stoma site. The IPAA case was included because it shared the same core technical concept of restorative pelvic surgery performed through prior stoma‐site access using the da Vinci SP platform. Accordingly, procedure‐specific outcomes should not be inferred from this small heterogeneous cohort.

Minimally invasive Hartmann's reversal and ileostomy takedown have been associated with reduced wound morbidity and enhanced recovery compared with open surgery [12, 13]. However, dense adhesions and deep pelvic work remain key drivers of operative difficulty and conversion [14]. Reported conversion rates in laparoscopic series range from approximately 10%–30%, and major morbidity rates range from 15% to 35% in contemporary series [4, 6, 15, 16]. Representative studies focusing on single‐access approaches, including single‐port laparoscopic surgery (SPLS) and SP robotic surgery, are summarised in Table S1, demonstrating a wide range of conversion and morbidity outcomes depending on access strategy and patient selection. In the present study, no open conversions occurred despite a reoperative cohort in which 83% of patients were classified as ASA class III, and major morbidity was limited to one Clavien–Dindo IIIa event. Although the small sample size and lack of a comparative arm preclude direct comparison, these findings suggest that the SP platform may provide sufficient technical control to extend a single‐site approach to selected high‐risk patients.

Previous robotic colorectal surgery series have mainly evaluated conventional multiport robotic platforms. Aliyev et al. compared the da Vinci Si and Xi systems in patients undergoing sphincter‐preserving total mesorectal excision for mid‐low rectal cancer and reported that the Xi system was associated with shorter operative time than the Si system [17]. Although that study did not evaluate the da Vinci SP platform, it provides a useful benchmark for conventional robotic colorectal surgery when interpreting the operative outcomes of the present series.

Single‐port laparoscopic stoma‐site reversal has been introduced to minimise additional incisions; however, restricted triangulation and instrument crowding may limit adhesiolysis and pelvic manoeuvres in adhesive fields [15, 18]. Previous SPLS studies using the stoma site as the access point have reported acceptable feasibility but variable morbidity and conversion rates, as shown in Table S1 [15, 19, 20, 21]. In this context, the articulated instrumentation and stable three‐dimensional visualisation of the SP robotic system may mitigate some of these constraints, particularly during adhesiolysis and intracorporeal reconstruction [15, 18, 22, 23, 24]. While prior single‐port approaches have primarily relied on laparoscopic platforms or abdominal single‐site access, the integration of a robotic SP platform with a stoma‐site access strategy represents a distinct technical evolution [15].

A key technical concept of this approach is the use of the prior stoma site as the primary access point, which may reduce the need for additional abdominal access and adhesiolysis solely for port placement. The following stoma mobilisation and transection, direct assessment of the peristomal space facilitates safe establishment of the pneumoperitoneum through the existing fascial opening. This access route may provide a favourable trajectory for pelvic dissection while avoiding additional abdominal wall trauma. Furthermore, the articulated instruments and flexible camera of the SP system facilitate adhesiolysis and intracorporeal reconstruction through a single access point. The multichannel single cannula may also mitigate external arm collisions and improve instrument maneuverability, which are common limitations of laparoscopic single‐port surgery. In addition, near‐infrared ICG perfusion assessment was readily performed using the SP platform, providing real‐time confirmation of anastomotic or pouch perfusion in all cases. Collectively, these technical features may facilitate the integration of access, adhesiolysis, and reconstruction through the previous stoma site in selected reoperative patients, although further comparative studies are required to determine whether these technical features translate into measurable clinical benefits.

Although no conversion, leakage or wound complications occurred, the two safety signals merit emphasis. First, an intraoperative small‐bowel injury occurred during adhesiolysis and required resection, underscoring that dense adhesions remain the dominant risk factor in reoperative abdomens, even with robotic assistance [25]. Second, one patient developed a postoperative intra‐abdominal abscess requiring percutaneous drainage (Clavien–Dindo IIIa). These events highlight that the platform may facilitate the technique, but does not eliminate adhesiolysis‐related risks, and careful patient selection and stepwise adhesiolysis remain essential. With respect to recovery, the median time to soft diet and length of stay were acceptable for a reoperative cohort; notably, one prolonged hospitalisation was attributable to institutional isolation for scabies rather than surgical morbidity and should not be interpreted as a procedure‐related delay.

The present approach should also be interpreted in the context of surgeon experience. The operating surgeon had substantial experience with minimally invasive colorectal surgery, conventional robotic surgery, and the da Vinci SP platform before adopting this stoma‐site approach. Such experience may be particularly important because this procedure involves adhesiolysis in a reoperative abdomen, pelvic dissection, intracorporeal reconstruction, and reduced triangulation through a single access route. Therefore, careful patient selection and sufficient experience with both colorectal reoperative surgery and the SP platform are required when introducing this technique.

This study was limited by its small sample size, retrospective design, and absence of a comparative control group, which precludes definitive conclusions regarding its superiority over laparoscopic or open approaches. The cohort included heterogeneous restorative procedures, and all procedures were performed by a single experienced robotic surgeon, introducing selection and performance biases. The follow‐up period was short, limiting the assessment of long‐term anastomotic durability and functional outcomes. Although console time was reported as a robotic‐specific operative metric, docking time was not prospectively recorded in a standardized manner and could not be analysed separately. Larger prospective and comparative studies are required to clarify optimal indications and long‐term outcomes. Nevertheless, this early experience provides clinically relevant insights into the feasibility of a single‐access restorative strategy in the reoperative abdomen, a setting traditionally associated with high technical difficulty and conversion risk [14]. These findings may inform patient selection and technical planning in centres that adopt the SP platform.

## 5 Conclusions

In this early experience, stoma‐site restorative colorectal surgery using the da Vinci SP system was technically feasible in carefully selected patients after emergency colorectal resection. However, the small sample size, heterogeneous procedures, and occurrence of adverse events preclude conclusions regarding clinical superiority or broad applicability. Further prospective comparative studies are required to clarify the safety, optimal indications, and role of this approach in restorative colorectal surgery.

## Author Contributions

**Hyeon Sik Jeong:** data collection, writing – original draft. **Dong Jin Park:** methodology, writing – original draft, writing – review and editing. **In Kyu Park:** methodology, writing – review and editing. **Songsoo Yang:** conceptualisation, methodology, writing – review and editing, supervision.

## Funding

The authors have nothing to report.

## Ethics Statement

Ethical approval was granted by the Institutional Review Board of the Ulsan University Hospital (2026‐02‐018).

## Consent

The requirement for informed consent was waived due to the retrospective design of the study.

## Conflicts of Interest

The authors declare no conflicts of interest.

## Supporting information


**Table S1:** Representative studies of single‐port minimally invasive stoma reversal and restorative colorectal surgery.

**Video S1:** Robotic hartmann’s reversal using the da Vinci SP System through the Previous stoma site. This video demonstrates robotic Hartmann’s reversal performed using the da Vinci SP robotic system through the previous stoma site without additional laparoscopic ports in a 44‐year‐old man with a history of open Hartmann’s procedure for diverticular perforation. The procedure includes detachment of the omentum from the abdominal wall, mobilization of the descending colon, extracorporeal anvil insertion, small‐bowel adhesiolysis from the pelvis, identification and mobilization of the rectal stump, re‐transection of the rectal stump, and intracorporeal colorectal anastomosis using the double‐stapling technique. The procedure was completed without drain placement or additional assistant ports, leaving only the previous stoma site as the abdominal incision.

## Data Availability

The data that support the findings of this study are available on request from the corresponding author. The data are not publicly available due to privacy or ethical restrictions.
