# Supplementary material for: Stoma‐Site Single‐Port Robotic Restorative Colorectal Surgery Using the da Vinci SP System: Technical Considerations and Early Outcomes
Source: Int J Med Robot. 2026 Oct 24;22(5):e70241. doi: 10.1002/rcs.70241 (PMC13695484; doi:10.1002/rcs.70241)
Supplement: Supplementary file 1 [file RCS-22-e70241-s002.docx]

**Supplementary Table 1.** Representative studies of single-port minimally invasive stoma reversal and restorative colorectal surgery

| **Study** | **Year** | **Approach** | **N** | **Conversion (%)** | **Major complication (%)** | **LOS (days)** |
| --- | --- | --- | --- | --- | --- | --- |
| Choi et al. [19] | 2015 | SPLS (stoma site) | 22 | 0% | 18.2% | 8 |
| Thambi et al. [20] | 2019 | SPLS (stoma site) | 56 | 8.9% | 8.9% | 4 |
| Keller et al. [15] | 2024 | SPLS vs SP robotics (abdominal site) | 50 vs 50 | 4% vs 0% | 12% vs 10% | 5 vs 4 |
| van Loon et al. [18] | 2022 | SPLS (stoma site) | 156 | 12.8% (open),  21.2% (multiport) | 28.2% | 4 |
| **Present study** |  | SP robotics (stoma site) | 6 | 0% | 16.7% | 6 |

**Abbreviations:** LOS, length of stay; SPLS, single-port laparoscopic surgery; SP, single-port

**Footnote**: Major morbidity was defined as Clavien–Dindo grade ≥ III where reported. Length of stay (LOS) is presented as reported in each study. Definitions of conversion varied across studies and included conversion to open surgery or addition of ports.
